# Navigating through HPV stigma: an intersectional lens on community engagement for vaccine acceptance in Pakistan

**DOI:** 10.1016/j.lansea.2026.100733

**Published:** 2026-02-24

**Authors:** Inayat Ali, Abida Sharif, Ayesha Qamar, Salma Sadique, Iffat Elbarazi

**Affiliations:** a Department of Anthropology, Fatima Jinnah Women University, Rawalpindi, Pakistan; b Department of Social and Cultural Anthropology, University of Vienna, Vienna, Austria; c Department of Community Medicine, Shifa Tameer-e-Milat University, Islamabad, Pakistan; d Department of Sociology, Fatima Jinnah Women University, Rawalpindi, Pakistan; e Department of Communication Studies, Fatima Jinnah Women University, Rawalpindi, Pakistan; f Department of Community Medicine, Peoples University of Medical and Allied Sciences, Nawabshah, Pakistan; g Institute of Public Health, College of Medicine and Health Sciences, United Arab Emirates University, Al Ain, United Arab Emirates

**Keywords:** Immunization, Sociocultural patterns, Geopolitics, Vaccine anxiety, Vaccine concerns, Infodemic

## Abstract

Human papillomavirus (HPV) vaccination represents a significant milestone in global public health efforts to prevent cervical cancer. Yet its implementation in some countries such as Pakistan reveals complex sociocultural and geopolitical challenges. Since the staged introduction of the HPV vaccine in Pakistan in 2025, coverage did not fully reach the targeted 9–14-year-old girls—falling short of the national goal of 90% by 2027. This commentary critically examines the intersectional barriers shaping HPV vaccine uptake in Pakistan, arguing that mistrust, stigma, and inequity are rooted in historical, gendered, religious, and geopolitical power dynamics. Applying an intersectional lens, the analysis demonstrates how vaccine resistance is influenced by moral anxieties surrounding adolescent sexuality, diverse religious interpretations, socioeconomic disparities, weak health infrastructure, and digital disinformation. Comparative insights from the United Arab Emirates and Saudi Arabia challenge assumptions that Muslim-majority contexts inherently resist HPV vaccination, highlighting instead the importance of socioculturally responsive strategies. The commentary proposes three policy implications for Pakistan: localized communication tailored to socio-cultural context, investment in social infrastructure and community engagement, and equity-sensitive monitoring frameworks. Addressing hesitancy requires recognizing community concerns as rational responses to lived experience rather than ignorance, for achieving equitable immunization and safeguarding girls’ health rights in comparable settings.

Vaccines are considered one of the proven preventive measures against various infectious diseases across the globe.^1^ Several vaccines are routinely recommended and delivered through national immunization systems, including Pakistan's essential Programme on Immunization (EPI).^2^ Rooted in the successful eradication of smallpox and ongoing efforts against measles, rubella, polio, coronavirus disease 2019 (COVID-19), and malaria, vaccination remains a cornerstone of global public health. In recent decades, vaccination against human papillomavirus (HPV) has become a growing focus of immunization programs worldwide, particularly in low- and middle-income countries, including Pakistan. This is due to its proven effectiveness in preventing cervical cancer—the fourth most common cancer among women globally—and its disproportionate burden in regions with limited access to screening and early treatment services.^3^

Pakistan initiated a staged introduction of the HPV vaccine for girls aged 9–14 years on September 15, 2025, beginning in three provinces and one administrative territory.^4^ Prior to this initiative, mass media and community channels were used to disseminate information highlighting the burden and preventability of cervical cancer. According to publicly reported program targets, the national goal is to reach 90% single-dose coverage among an estimated 13 million eligible girls between 2025 and 2027.^4^ By September 25, 2025, approximately 34% single-dose administrative coverage had been reported in implementing areas, although the precise denominators, reporting sources, and completeness of these figures require clearer documentation.^5^ This early shortfall raises various questions about the structural, geopolitical and socio-cultural factors that may hinder attainment of national targets.

Research has consistently identified Pakistan as a setting where vaccination coverage for several antigens remains below the recommended global benchmarks.^2^^,^^6^^,^^7^ Polio eradication efforts in Pakistan illustrate the complex interplay of sociocultural distrust, misinformation, and political insecurity that can impede vaccine acceptance. Some ethnographic work documents how rumors about vaccine safety became entangled with suspicions of Western interference, leading some communities to resist house-to-house vaccination teams and view them as agents of broader geopolitical agendas rather than public health providers.^8^, ^9^ These dynamics were exacerbated by mistrust of government and international partners engaged in immunization campaigns.^8^^,^^9^ Contemporary analyses reveal that chronic governance challenges, including insecurity in high-risk districts, weak health infrastructure, and low community engagement, have further complicated polio efforts despite substantial investment.^10^ Hussain and colleagues also note that beyond logistical obstacles, polio vaccination campaigns have at times been perceived as coercive or externally imposed, deepening skepticism among families already facing structural marginalization.^11^ Together, these studies show that low vaccination uptake in Pakistan cannot be explained by information deficits alone; rather, vaccination programs must contend with long-standing issues of (mis-)trust, locally circulating narratives about intent, and the broader socio-cultural and geopolitical context in which communities interpret public health interventions.

Persistent transmission of poliovirus, despite decades of effort, illustrates the scale of trust and access-related challenges surrounding immunization programs. As mentioned earlier, some parents delay or refuse vaccination despite biomedical availability because of intersecting socio-cultural, economic, and geopolitical factors.^2^, ^8^, ^9^ The gap between scientific promise and social reality signals that mistrust, misinformation, and inequality continue to significantly shape vaccine acceptance.^9^^,^^12^ Understanding these barriers requires more than communication strategies; it warrants attention to how multiple sociocultural factors—gender, religion, class, geography, and technology—intersect to produce differentiated experiences of health.

Using an intersectional analytical framework, vaccine-related concerns in Pakistan appear not as a single attitude or uniform “hesitancy” but as a constellation of forces embedded in power relations. Gendered moral expectations are central. Because the HPV vaccine protects against a virus that can be transmitted through sexual relations, some parents fear that consenting to vaccination implies recognition of adolescent sexuality.^13^ In a society where women's modesty is closely tied to family honor, health messages can unintentionally collide with deeply held cultural values. Religious interpretations add further complexity.^14^ While many religious leaders have publicly endorsed vaccination as preventive care consistent with Islamic ethics, others question its necessity or frame it as externally driven, creating moral ambiguity that health authorities may underestimate. Where evidence is limited, these dynamics should be understood as plausible inferences that require further Pakistan-specific empirical study.

The experiences of the United Arab Emirates (UAE) and the Kingdom of Saudi Arabia (KSA) offer contextually relevant but non-transferable insights for Pakistan. Although neither country can be considered an early global pioneer in HPV vaccine introduction, their recent experiences demonstrate that HPV vaccination is feasible in Muslim-majority settings.^15^ Differences in governance capacity, financing mechanisms, school-entry enforcement, and migrant population management mean that their models cannot be directly replicated in Pakistan. However, elements such as school-based delivery platforms, centralized procurement, and engagement with faith leaders may be adaptable, while others are not. In the UAE, the inclusion of large South Asian migrant communities within school-based vaccination programs demonstrates how culturally sensitive and well-structured initiatives can enhance acceptance and uptake.^15^ Ensuring HPV vaccine acceptance and uptake therefore requires locally grounded implementation strategies aligned with global public health standards rather than wholesale policy transfer.

Socioeconomic position and geography further shape access to reliable information. Families in rural or low-income settings frequently rely on informal communication networks that are more susceptible to rumours and conspiracy theories. Health infrastructure in peripheral districts remains weak, and documented shortages of female frontline health workers may undermine trust, particularly for services targeting adolescent girls. Meanwhile, digital platforms, though powerful channels for outreach, also amplify competing narratives through social-media echo chambers. Rather than assuming that marginalised populations alone are exposed to negative messaging, as mentioned previously, studies from Pakistan suggests that vaccine misinformation circulates widely across mainstream, digitally connected groups, highlighting the need for nuanced framing.

Against this background, suspicion that the HPV vaccine represents a “Western plot” does not arise in a vacuum. It reflects a historical memory of medical interventions in which colonial or international actors exercised power without accountability. Historical scholarship documents episodes of medical injustice in multiple settings, where women were subjected to coerced sterilization and unethical reproductive health interventions.^16^ As mentioned earlier, the history of resistance to polio vaccination widely documented in Pakistan may be an even more proximate analogue.^8^, ^9^ These cases are not presented as direct causes of contemporary HPV concerns in Pakistan, but as illustrative examples of how collective memory of medical injustice can shape trust in public health systems. Recognising this context, thus, is essential for designing communication that respects communities’ historical consciousness rather than dismissing it as ignorance.

Coverage statistics alone cannot capture whether access is equitable or whether trust has been rebuilt in communities historically excluded from health decision-making. Disaggregated coverage is essential but insufficient without attention to voice, agency, and accountability. Intersectional public-health practice calls for gender-responsive policies that empower mothers and daughters as informed decision-makers, for constructive engagement with religious authorities rather than assumed confrontation, and for digital governance that curbs misinformation while expanding inclusive participation.

For Pakistan, three interrelated implications emerge that extend beyond biomedical logic and highlight the socio-cultural and geopolitical nature of vaccination.

First, vaccine communication must be culturally situated rather than centrally scripted. Public health messaging often assumes that information deficits drive hesitancy, yet anthropological studies shows that health decisions are embedded within local moral worlds. HPV vaccination campaigns must therefore engage with the symbolic meanings attached to girlhood, sexuality, purity, and family honor. This requires not only translation across languages but also translation across worldviews—co-creating narratives with community elders, mothers, youth, and professional associations such as national gynecological societies that have endorsed the vaccine rollout.

Second, investment in social infrastructure must be recognized as a form of public health intervention. It is now well established that trust is not produced by messages alone but by relationships. Strengthening vaccination uptake demands long-term engagement with communities through relational networks: training and retaining female frontline health workers, building partnerships with religious authorities, and supporting community-based media that enable dialogue rather than top-down persuasion. Recent work on the Vaccine Trust Framework provides a useful conceptual and empirical basis for this approach.^17^ Drawing on mixed-method approach in low- and middle-income settings, the framework demonstrates that trust in vaccination is multidimensional, encompassing expectations of health system promise, experiences of service delivery, beliefs about vaccine benefits, and perceptions of how vaccines are delivered in practice. Importantly, it shows that gaps in trust often reflect lived experiences of health systems rather than information deficits alone. Digital literacy initiatives should accompany vaccination campaigns to counter algorithm-driven rumor economies. These social investments are not peripheral—they are fundamental to rebuilding confidence in state health systems historically perceived as distant or extractive. Together, these insights suggest that HPV vaccination strategies in Pakistan should be informed by systematic assessment of trust domains, using validated tools to identify where trust is most fragile and to guide context-specific interventions rather than generic messaging.

Third, monitoring frameworks must shift from headline coverage targets to equity-sensitive indicators.^18^ Rather than focusing solely on national averages, an equity-centered approach acknowledges that access to vaccines is shaped by structural conditions such as class, gender, geography, and mobility.^19^^,^^20^ In the Pakistani context, a pragmatic minimum disaggregation set—such as urban–rural residence, district, school enrollment status, and maternal education—may be more feasible and ethically appropriate than ethnicity-based reporting, while still illuminating systematic gaps. Such an approach reframes vaccination not merely as a logistical challenge but as a question of social justice, accountability, and the right to health.

The HPV vaccination campaign is thus more than a public-health intervention; it is a social negotiation over trust, autonomy, and memory. Acknowledging the rationality behind mistrust—especially when it is informed by histories of medical inequality—does not legitimise conspiracy theories; rather, it situates them within lived experiences that policymakers must address to formulate and implement policies. Dealing with HPV-related stigma requires structural empathy, defined here as the capacity of health systems to recognize historical disparities, attend to social inequities, and act with accountability. Only through such inclusive and historically grounded approaches can Pakistan and other countries navigating similar terrains fulfil the vaccine's promise—to protect every girl's right to a healthy future.

## Data sharing statement

Data sharing not applicable—no new data generated.

## Declaration of interests

There are no known conflict of interests that might have affected this study or launch of HPV roll out.
